# Management of Sickle Cell Disease: A Review for Physician Education in Nigeria (Sub-Saharan Africa)

**DOI:** 10.1155/2015/791498

**Published:** 2015-01-18

**Authors:** Ademola Samson Adewoyin

**Affiliations:** Department of Haematology and Blood Transfusion, University of Benin Teaching Hospital, PMB 1111, Benin City, Edo State, Nigeria

## Abstract

Sickle cell disease (SCD) predominates in sub-Saharan Africa, East Mediterranean areas, Middle East, and India. Nigeria, being the most populous black nation in the world, bears its greatest burden in sub-Saharan Africa. The last few decades have witnessed remarkable scientific progress in the understanding of the complex pathophysiology of the disease. Improved clinical insights have heralded development and establishment of disease modifying interventions such as chronic blood transfusions, hydroxyurea therapy, and haemopoietic stem cell transplantation. Coupled with parallel improvements in general supportive, symptomatic, and preventive measures, current evidence reveals remarkable appreciation in quality of life among affected individuals in developed nations. Currently, in Nigeria and other West African states, treatment and control of SCD are largely suboptimal. Improved knowledge regarding SCD phenotypes and its comprehensive care among Nigerian physicians will enhance quality of care for affected persons. This paper therefore provides a review on the aetiopathogenesis, clinical manifestations, and management of SCD in Nigeria, with a focus on its local patterns and peculiarities. Established treatment guidelines as appropriate in the Nigerian setting are proffered, as well as recommendations for improving care of affected persons.

## 1. Introduction

Sickle cell disease (SCD) is one of the most common genetic diseases worldwide and its highest prevalence occurs in Middle East, Mediterranean regions, Southeast Asia, and sub-Saharan Africa especially Nigeria [1, 2].

SCD is a chronic haemolytic disorder that is marked by tendency of haemoglobin molecules within red cells to polymerise and deform the red cell into a sickle (or crescent) shape resulting in characteristic vasoocclusive events and accelerated haemolysis. It is inherited in an autosomal recessive fashion either in the homozygous state or double heterozygous state. When inherited in the homozygous state, it is termed sickle cell anaemia (SCA). Other known SCD genotypes include haemoglobin SC disease, sickle beta plus thalassaemia, and sickle beta zero thalassemia (which has similar severity with sickle cell anaemia), haemoglobin SD Punjab disease, haemoglobin SO Arab disease, and others.

In Nigeria, SCD forms a small part of the clinical practice of most general duty doctors, as there is gross absence of dedicated sickle cell centres. Thus, it may be difficult to keep abreast of current knowledge and practices in the treatment of SCD. The purpose of this paper therefore is to provide a comprehensive and concise review of SCD and its management for physician education in Nigeria. Particular attention is given to its local epidemiology, clinical phenotypes and complications, current treatment guidelines, practice challenges, and recommendations for improved care. Relevant literatures and local references including clinical studies, reviews, and texts were gathered, summarized, and presented in this paper.

## 2. Epidemiology

About 5–7% of the global population carries an abnormal haemoglobin gene [3, 4]. The most predominant form of haemoglobinopathy worldwide is sickle cell disease. The greatest burden of the disease lies in sub-Saharan Africa and Asia [5].

The prevalence of sickle cell trait ranges between 10 and 45% in various parts of sub-Saharan Africa [6–8]. In Nigeria, carrier prevalence is about 20 to 30% [9, 10]. SCD affects about 2 to 3% of the Nigerian population of more than 160 million [9]. Recent estimate from a large retrospective study by Nwogoh et al. in Benin City, South-South Nigeria revealed an SCD prevalence of 2.39% and a carrier rate of about 23% [11].

## 3. Brief History and Genetic Origin of SCD

In 1874, Dr. Horton, a Sierra Leonian medical Doctor, reportedly gave the first description of clinical symptoms and signs which is now referred to as sickle cell disease [12]. Herrick, a Chicago physician, also gave a formal description of the disease in 1910 when he observed abnormal sickle shaped red cells in the blood of a dental student from West Indies who had anaemia [13]. In 1927, Hahn and Gillespie observed that sickling of red cells was associated with conditions of low oxygen tension. In 1949, Linus Pauling and colleagues demonstrated that haemoglobin in these patients was different from normal subjects using protein electrophoresis [14]. However, Venon Ingram and J. A. Hunt in 1956 sequenced the sickle haemoglobin molecule and showed that the abnormality was due to valine substitution for glutamate on the 6th position of the sickle beta-haemoglobin gene. Marotta and coworkers in 1977 showed that the corresponding change in codon 6 of the beta-globin gene was GAG to GTG [14]. Since then, further insights have been gained into understanding the origin, complex pathophysiology, and treatment of the disease through molecular biology techniques.

Africa and Asia are considered as the birthplace of the sickle cell mutation. Sickle cell disease is believed to be a consequence of natural mutation of the beta-globin gene (HBB) affecting the gametes and transferred to subsequent generations. Using restriction fragment length polymorphism analysis, four main African haplotypes and one Asian haplotype of the beta-globin chain genes have been characterized and are believed to originate differently in these regions. The main African haplotypes include Senegal, Benin, Bantu (central-African republic), and Cameroon haplotype [15–18]. The Bantu haplotype is associated with the most severe disease phenotype while the Asian (also called Arab-Indian) haplotype is associated with a mild phenotype [19].

SCD is found in other parts of the world including USA and Europe due to migration and interracial marriages [5, 20]. The high prevalence of SCD in sub-Saharan Africa has been attributed to survival advantage conferred by the sickle cell trait against* Plasmodium falciparum*. Resistance of individuals with sickle cell trait to* Plasmodium falciparum *creates a selective pressure that has maintained the sickle cell gene within human populations in malaria endemic regions like sub-Saharan Africa. This phenomenon is termed balanced polymorphism [21, 22].

## 4. Aetiopathogenesis of Sickle Cell Disease

SCD is a qualitative haemoglobinopathy resulting from a structural change in the sequence of amino acids on the beta globin chain of the haemoglobin molecule due to a point mutation. The sickling mutation causes a single base change from adenine to thymine on the 17th nucleotide of the beta globin chain gene (HBB). This invariably translates into substitution of valine for glutamate on the 6th amino acid of the beta globin chain. The abnormal biochemistry of this mutant haemoglobin induces polymerization of Hb S molecules within the red cells, so called sickling. On the sickle haemoglobin, the glutamate protein molecule, which is hydrophilic, polar, and negatively charged, is replaced by a less polar, hydrophobic, neutral amino acid, valine. Under deoxy conditions, the abnormal valine residue causes intraerythrocytic hydrophobic interaction of sickle haemoglobin tetramers, leading to their precipitation and polymer formation, so called gelation [23]. Eventually, all cytosolic haemoglobin molecules precipitate into seven (one inner and six outer) double strands with cross-links which are called tactoids. Upon reoxygenation, unsickling occurs and the red cell assumes its normal shape. However, repeated sickling and unsickling of the red cell damages the red cell membrane, due to herniation of sickle haemoglobin polymers through the cytoskeleton, thus rendering the red cell permanently sickled. These appear as irreversibly sickled cells (ISCs) on peripheral blood cytology.

The kinetics of red cell sickling is highly heterogenous. Several variables are known to affect the rate and degree of sickling of the red cells. Intracellular dehydration of sickle red cells increases mean cell haemoglobin concentration (MCHC) [14]. Higher MCHC favours sickling. As such, very high Hb S level of about 80 to 90% seen in the homozygous disease is associated with a worse disease while the presence of alpha thalassemia (one or two gene deletions) ameliorates the disease. Another variable is the presence of other interacting nonsickle haemoglobin. Of note is fetal haemoglobin (Hb F). Higher proportion of Hb F is associated with mild disease. When present, high levels of Hb F are uniformly dispersed within the red cell and it retards the sickling process. Thus, coinheritance of sickle haemoglobin with hereditary persistence of fetal haemoglobin (HPFH) is associated with mild disease [24]. Similarly, this advantage is positively utilized through clinical use of fetal haemoglobin inducing drug such as hydroxyurea. Vascular beds that have intrinsically sluggish venous outflow such as bone marrow, spleen, or inflamed tissues are at higher risk of infarctive events due to prolonged microvascular transit time [25]. Whenever and wherever microvascular transit time becomes longer than sickling delay time, sickling and vascular occlusion become imminent. Intracellular pH is another important variable. With acidosis, the haemoglobin molecules give off their oxygen more readily and sickling occurs more readily.

Repeated sickling of the red cell induces cellular injury which has been shown to activate membrane ion channels such as the Gardos pathway (calcium gated potassium channels) and KCL cotransporter [25]. There are influx of calcium ions and efflux of potassium and water, hence intracellular dehydration. High intracellular calcium levels provoke activity of proteolytic enzymes such as phospholipases and proteases causing the digestion of membrane phospholipids and proteins, respectively. Subsequently, there is perturbation of the membrane lipids with exteriorization of lipids such as phosphatidyl serine and ethanolamine which are normally located in the inner leaflets of the membrane lipid bilayer [26].

The diverse clinical heterogeneity of SCD is related to two main pathogenetic processes: chronic haemolysis and high viscosity/vascular occlusion. Infarctive events in SCD result from erythrostasis caused by rigid sickled cells in various vascular beds especially organs with sluggish blood flow such as the spleen and the bone marrow. Capillaries are about 2-3 microns in diameter. Sickle cells due to loss of flexibility are unable to transit the microvasculature, hence vessel occlusion. Aside from these mechanistic processes, sickle cells are also shown to exhibit increased adhesiveness to vascular endothelium, leucocytes, platelets, and themselves [27–29]. Sickle reticulocytes are even more adhesive to the endothelium than sickle discocytes [30]. Molecular interactions between the red cells and the vascular endothelium include CD36 and thrombospondin, VLA4 and VCAM-1, respectively [30, 31]. Fibronectin and von Willebrand factor are also involved in these interactions. Currently, it is known that increased adhesiveness of different cellular surfaces with formation of heterocellular aggregates is believed to propagate the phenomenon of vascular occlusion, especially in the postcapillary venules [26]. Also note that destruction of red cell membrane causes exposure of membrane proteins, thereby inciting autoantibody formation. These antibodies such as IgG anti-band 3 antibodies are believed to promote erythrophagocytosis [25].

Episodic microvascular occlusion in sickle cell disease even in steady state results in ischaemic-reperfusion injury which sets the stage for an increased inflammatory tone, thus significant elevations in total leucocyte counts, platelet counts, and positive serum acute phase reactants. Even in steady state, SCD is a chronic inflammatory condition; the attendant inflammation induced oxidative stress further contributes to progressive tissue damage [32, 33]. The leucocytes in SCD also express higher levels of L-selectins and also have stimulated adhesiveness. Increased adhesiveness coupled with phosphatidyl serine exposure on the red cell surface makes SCD a procoagulant and a hypercoagulable state [34].

Erythrocyte lifespan in SCD averages about 16–20 days in contrast to about 100–120 days in normal state [35, 36]. Haemolysis in sickle cell disease is both extravascular and intravascular. Abnormal shape of the ISCs creates an abnormal rheology, associated with heightened clearance by the reticuloendothelial system. Because of their increased fragility and reduced deformability, some red cells undergo intravascular haemolysis. High plasma haemoglobin level is associated with low haptoglobin levels and high levels of lactate dehydrogenase (LDH), arginase-1, and AST [37]. Plasma haemoglobin is an avid scavenger of nitric oxide (NO). High plasma haemoglobin levels resulting from chronic haemolysis reduce NO bioavailability. Normally, NO relaxes the endothelium and maintains vascular tone (vasodilator) [37]. Low circulating levels of NO propagate vasospasm which is observed even in large vessels in SCD. Contributing to this is dysfunction of endothelial nitric oxide synthetase [37]. This is the underlying basis of vasculopathic complications such as cerebrovascular diseases, priapism, pulmonary hypertension, and chronic leg ulcers. Arginase 1 is normally involved in formation of urea in protein excretion. Accelerated haemolysis of red cells leads to higher levels of arginase 1. As such, more ornithine is produced, further depleting plasma arginine levels. Excess ornithine is channeled to alternate pathways which produce excess prolenes and polyamines. These byproducts promote endothelial smooth muscle proliferations, further narrowing the vascular chamber [38]. Chronic haemolysis results in excess breakdown of haemoglobin molecules and high levels of bilirubin, which is associated with formation of bilirubin pigment stones in the gall bladder (cholelithiasis).

Some authorities have attempted to categorize SCD patients into two clinical subphenotypes based on the overriding pathogenic process [25, 39]. Clinically, there is some degree of overlap between the two groups. Some patients experience more of viscosity/vasoocclusive complications and tend to have higher baseline haematocrit levels. Others experience more of vasculopathic complications due to more intense haemolysis associated with a lower baseline haematocrit [14, 39].

## 5. Clinical Phenotypes and Complications in Sickle Cell Disease

There is marked intraindividual and interindividual variability in SCD. Clinical heterogeneity of the disease has been explained by both genetic and environmental factors. Known genetic factors contributing to variations in clinical severity of the disease include the pattern of sickle cell inheritance, nature of b-globin haplotype, Hb F level, and FCP loci [15]. Other modulators of the disease include presence of alpha thalassemia and other probable genetic influences as well as environmental factors such as access to optimal health care, ambient living conditions, and availability of finance [15].

*Physical Effects of Sickle Cell Disease*. Body habitus in SCD ranges from a normal build to a tall, lanky physique depending on the clinical severity. Other physical changes include prognathism, arachnodactyly, and increased AP chest diameter (barrel chest) [40, 41]. In childhood, sickle cell patients may be shorter or smaller than normal. Puberty is often delayed but considerable growth takes place in late adolescence such that adults with sickle cell anaemia are at least as tall as normal [14]. However, adults that have suffered vertebral infarction and collapse may be shorter than normal. Many of these physical changes are due to the chronic hypoxaemia associated with severe anaemia. Severe haemolysis in infancy causes marrow hyperplasia of the skull and facial bones, resulting in frontal bossing, prognathism, or malocclusion [42]. The abnormal facies results from extension of the marrow into the cortical bone causing widening of the diploe spaces and thinning of the bone cortex. The chronic haemolytic process is associated with pallor, jaundice, splenomegaly in early childhood.

### 5.1. Acute Sickle Syndromes/Complications

#### 5.1.1. Bone Pain Crisis (BPC)

BPC is the most consistent and characteristic feature of SCD [43]. The pain results from activation of nociceptive afferent nerve endings in the ischemic bones. Commonly affected bones include the long bones such as femur and humerus, vertebrae, pelvis, ribs, and sternum [24]. Multiple sites may be involved. An early manifestation of bone infarction is the hand and foot syndrome. This is characterized by dactylitis involving the small bones of the hand or foot, marked by diffuse swelling over the involved area. It often resolves spontaneously within one to two weeks and is rare after 2-3 years of life. Frequency of bone pain crisis is higher in patients with homozygous sickle cell disease, low Hb F, and higher baseline haemoglobin. It is said to be more common in young adults but its frequency tends to wane at older ages. Pain episodes vary in intensity and tend to resolve within a few days. In about 57% of cases, no precipitating factor is identified [44]. However, known precipitants include exposure to cold, dehydration, intercurrent infections such as malaria, physical exertion, tobacco smoke, alcohol use, hard drugs, high altitude, hypoxic conditions, physical pain, pregnancy, hot weather, emotional stress, or onset of menses [32, 45]. Suggested treatment guideline for uncomplicated BPC is presented as follows.

*Treatment Guidelines for Bone Pain Crisis*

- Principles of treatment include adequate analgesia, hydration, warmth, prophylactic or therapeutic antibiotics if pyrexial after necessary culture samples are taken, as well as oxygenation if hypoxic (Sp O_2_ < 90%) [45–48].
- Oral hydration must be adequate with at least 1.5 L/m² of water based fluid per day in children and 60–70 mL/kg in adults. If parenteral, not more than 1.5 times maintenance is given in order to prevent volume overload considering baseline anaemia in most patients [49, 50].
- Patients and parents should be encouraged to keep a stock of simple analgesics at home in event of a painful episode. However, mild to moderate pain that does not succumb to home-based oral analgesia and hydration within 2 days requires hospitalization.
- Analgesia should be commenced within 15 to 30 minutes of presentation in the emergency room or day hospital. Effective analgesia should be achieved within 1 hour. There should be an ongoing assessment of analgesic efficacy every 30 minutes until pain is controlled, thereafter every 2 hours [49, 50].
- Treatment should be individualized. The choice of analgesia depends on the severity of the pain and the patient's prior analgesic needs/history.
- Nonopioids such as simple paracetamol and NSAIDS (nonsteroidal anti-inflammatory drugs) may be used in mild VOC. Weak opioids such as tramadol and DF118 (dihydrocodeine) are used for moderate pain while severe pain requires stronger opioids/narcotics such as morphine [45, 46].
- Adjuvants for pain control help in achieving better analgesia. They may include mild sedatives such as promethazine or diazepam. Combination of paracetamol or NSAIDS with opiates gives better analgesia because of their synergistic actions. Oversedation should be avoided. Laxatives should be prescribed for prevention and treatment of constipation, a side effect of opioid use.
- More than five to seven days of sequential NSAID use should be avoided to reduce the risk of peptic ulceration and GIT haemorrhage. Also, NSAIDS are potentially nephrotoxic and are better avoided in established renal disease.
- Severe VOC requires parenteral opioid analgesia and hydration in a hospital setting. The dose of the analgesia should be titrated with the severity of the pain until adequate control is achieved in a fixed dose schedule (FDS), interspersed with short-acting agents for breakthrough pains.
- Prophylactic incentive spirometry is recommended for prevention of acute chest syndrome especially in BPC involving the chest wall. In the absence of a spirometer, 10 deep breaths every 2 hours while awake between 8 a.m. and 10 p.m. are an alternative.
- If pain persists, patient controlled analgesia (PCA) should be considered where available. PCA is sparsely available in Nigeria, except in very few private facilities. PCA reduces the risk of pain undertreatment.
- Short-acting opioids in clinical use include tramadol, morphine, hydrocodeine, hydromorphine, fentanyl, oxymorphine, and oxycodeine. Longer acting opioids include methadone and slow release preparations of tramadol, morphine, and oxycodeine. Access to a wide range of opioids may not always be readily available in Nigeria; however, the available ones should be used.
- In difficult cases, where pain is unremitting after 48 hours of well conducted analgesia, exchange blood transfusion (EBT) may be offered [51].
- Hospitalization for severe BPC occurring on 3 or more occasions per year is an indication for initiation of hydroxyurea therapy or chronic transfusion therapy in patients that are intolerant of hydroxyurea.
- Pain in SCD is majorly nociceptive in origin. However, it may also have a neuropathic component marked by tingling/burning sensation or numbness. In such cases, drugs such as pregabalin or carbamazepine will be useful [46].
- Pain control in SCD is essentially pharmacologic. However, nonpharmacologic measures such as physical therapy with heat or ice packs, relaxation, distraction, music, menthol rub, meditation, and transcutaneous electrical nerve stimulation (TENS) are also helpful [46].

Treatment of acute sickle cell pain in a dedicated day hospital is associated with better outcome due to prompt triage and familiarity with analgesic needs of individual patients, hence reduced risk of pain undertreatment. Invariably, there is reduction in overall patient admission rates and better outcomes compared to emergency room settings [52–54]. However, most institutions in Nigeria lack daycare settings for management of sickle cell crisis.

Furthermore, it is important to note that BPC may be complicated by a concurrent hyperhaemolytic crisis with resultant acute severe anaemia or may even progress to acute chest syndrome or multiorgan failure syndrome (MOFS). MOFS is defined as sudden onset, severe organ dysfunction simultaneously involving at least two major organ systems (such as the liver, lung, and kidney) in the setting of an acute sickle cell crisis. MOFS is partly explained by significant vasoocclusive events in vital organs with major functional compromise and organ failure [55]. This life-threatening complication requires immediate intensive care and a multispecialist attention including the intensivists, nephrologist, hepatologist, respiratory physicians, and others.

#### 5.1.2. Acute Abdominal Pain

Acute abdominal pain in SCD may be due to sequestration crisis, vasoocclusion of mesenteric vessels, gall bladder/biliary tract disorders, or other non-SCD specific causes. In a Nigerian study by Akingbola et al., the aetiology of acute abdominal pain in a population of adult Nigerian SCD patients presenting in a tertiary facility were found to include SCD related complications such as abdominal VOC and acute cholecystitis, as well as other infective causes such as cystitis, gastroenteritis, appendicitis, and bowel obstruction [56]. About 38% of the cases were due to abdominal infarction/crisis [56]. In Ile-Ife, Akinola et al. observed abdominal pain due to VOC in 26% of sickle cell anaemia patients [57]. Microvascular occlusion may involve the mesenteric bed causing ischaemic abdominal pain. Abdominal pain of presumed vasoocclusive origin is termed abdominal crisis. Girdle syndrome, otherwise called mesenteric syndrome, is a rare complication owing to extensive collateral blood supplies to the mesentery and bowel wall [58]. Girdle syndrome or mesenteric syndrome is said to be present when there is an established paralytic ileus, which may be associated with vomiting, silent distended abdomen, dilated bowel loops, and air-fluid levels on abdominal radiography. Typically, a patient with girdle syndrome presents with generalized abdominal pain and rarely in shock if there is massive bowel gangrene (third space losses). Other abdominal findings may include localized or rebound tenderness, board-like rigidity, and lack of movement on respiration. Abdominal radiography and ultrasound scan are helpful as well as investigations to rule out differentials such as pancreatitis, acute appendicitis, cholecystitis, biliary colic, splenic abscess, ischaemic colitis, and other forms of acute abdomen. Intravenous hydration, analgesia, and antibiotics are indicated. For an established ileus, NPO (nothing by mouth) should be commenced, as well as nasogastric aspiration, if there is vomiting. Urgent surgical opinion should be sought. Exchange blood transfusion may be necessary in mesenteric (girdle) syndrome. Biliary/gall bladder anomalies commonly observed in SCD include cholelithiasis and biliary sludge [59]. Recent studies among Nigerian patients observed an age-related prevalence of about 5 to 10% for cholelithiasis [60, 61].

#### 5.1.3. Visceral Sequestration Crisis

Infants and children less than 7 years are at greatest risk of sequestration crisis especially splenic sequestration. Children above this age group and adults are at less risk of splenic sequestration because the spleen tends to become fibrotic with repeated infarctions and cannot enlarge [24]. However, in haemoglobin SC disease, older children and adults can experience sequestration crisis. If not corrected rapidly, acute sequestration results in hypovolaemia, severe anaemia, and possibly death. A typical patient is irritable with rapidly enlarging spleen or liver and pain in the upper abdomen. Features of acute anaemia include worsening pallor, generalized weakness, and tachycardia. Early presentation in the hospital and close monitoring are important. Blood transfusion is necessary if haemoglobin level falls 2 g/dL below steady state haemoglobin levels or evidence of cardiac decompensation. A major sequestration crisis is defined by haemoglobin level below 6 g/dL. There is risk for recurrence; therefore parents and caregivers must be taught how to examine the child's spleen regularly and report any abnormal finding to the physician immediately. Splenectomy is recommended after the second episode in children above two years of age. For children below 2 years of age, chronic blood transfusion should be offered [62]. Majority of SCD sequestration crisis involves the spleen. Though less common, the liver and lymph nodes may also be sites of sequestration in SCD.

#### 5.1.4. Aplastic Crisis

Aplastic crisis usually occurs in those less than 16 years of age. It is commonly caused by parvovirus B19 infection which causes transient selective suppression of erythroid progenitors. In Nigeria, prevalence of parvovirus B19 infection is shown to be similar among sickle cell patients and the general population [63]. In normal subjects, parvo-virus B19 infection is asymptomatic. However, in patients with chronic haemolytic anaemia such as SCD, parvoviral infection is potentially devastating. Reticulocytopenia often lasts about 7 to 10 days, followed by spontaneous remission with reticulocytosis. Aplastic crisis may follow a recent upper respiratory tract infection and the patient may have flu-like symptoms such as headache, mild fever, and lethargy. Other findings include pallor and worsening anaemia. The condition is self-limiting. Treatment is to give red cell transfusion support until erythroid activity resumes.

#### 5.1.5. Worsening (Acute) Anaemia in SCD

Baseline haemoglobin level in sickle cell disease ranges between 6 and 9 g/dL [64]. A study among adult SCD patients in Lagos by Akinbami et al. shows mean steady state haemoglobin levels of 7.92 ± 1.49 g/dL [65]. Average haemoglobin concentration of an SCD patient over a minimum of 4 weeks is considered steady state (or stable) haemoglobin level in the absence of any form of crisis in the preceding three months. Anaemia in SCD is usually well compensated. Occasionally, some patients have high steady state haematocrit level above 10 g/dL and they tend to present with more vasoocclusive complications. This has been termed high haematocrit syndrome [26]. Therapeutic phlebotomy when haemoglobin level is higher than 12 g/dL may benefit such patients [59]. However, in most other patients, significant decline of more than 2 g/dL below steady state level has functional consequences. Untreated severe anaemia is symptomatic and may precipitate heart failure. Causes of worsening anaemia in sickle cell disease may include hyperhaemolysis from any cause, aplastic crisis, megaloblastic crisis, iron deficiency, haemorrhage, renal failure, sequestration crisis, and extreme bone marrow necrosis. Treatment requires both definitive and supportive care. If patient shows signs of cardiac decompensation, blood transfusion should be given. The cause of worsening anaemia should be sought and treated accordingly.

#### 5.1.6. Cerebrovascular Disease (CVD)

CVD is a significant cause of morbidity and mortality in sickle cell disease [56]. CVD or stroke refers to a sudden onset focal or global neurologic deficit of vascular origin lasting more than 24 hours. It may be ischaemic or haemorrhagic. TIA or stroke occurs in 25% of patients with sickle cell disease. Overt stroke occurs in 10 to 15% of homozygous patients under the age of 10 years [66–68]. The prevalence of overt stroke among SCA children in Port Harcourt, Nigeria, is reported as 4.3% [69]. In Abuja, Nigeria, Oniyangi et al. reported a stroke prevalence of 5.2% among SCD children seen in a tertiary center [70]. The risk of CVD is higher in those with low baseline haemoglobin, low fetal haemoglobin, high white blood cell count, and high systolic blood pressure. CVD is rare in infants. Incidence of CVD is higher in Hb SS disease compared with SC disease, S/b+thalassemia, and S/b zero thalassemia [36, 59].

Infarctive CVD is commonest and occurs in patients aged less than 20 years and older than 30 years (peak incidence: 10–15 years) [71]. Infarction is often associated with stenosis or occlusion of affected vessels most commonly the distal internal carotid, proximal middle cerebral, and anterior cerebral arteries. The patient may present with antecedent history of TIA or seizures, which eventually progresses to an overt stroke, characterized by hemiparesis, speech, or visual impairment, or even coma if immediate therapy is not instituted. Suggested guideline on acute and long-term treatment of an ischaemic stroke in SCD is provided as follows.

*Treatment Guidelines for Sickle Cell Ischaemic Stroke*

- After initial evaluation of patient's airway, breathing, and circulation (ABC of resuscitation), further stabilization should be pursued through prevention and control of hypoxaemia, hypotension, hyperthermia, and glycaemic imbalance, which would worsen the cerebral insult.
- Presence of seizures should be controlled with appropriate anticonvulsants. Prophylactic antiseizure therapy is not necessary.
- Urgent noncontrast CT/MRI is required to distinguish haemorrhage and infarction. This important distinction has to be made early, as this will impact subsequent therapeutic decisions.
- In the early stage of brain ischaemia (<3 hours), cranial CT may be negative or show only subtle inconclusive signs. Magnetic resonance imaging (MRI) provides better details but should be deferred until treatment has been initiated.
- Early institution of exchange transfusion is crucial to improving treatment outcome. EBT should be targeted at reducing sickle Hb level below 30%. Simple transfusions may be offered in the interim while EBT is being planned. Simple transfusions at 10–15 mL per kg red cells reduce sickle haemoglobin levels to about 60%.
- Adequate hydration not more than 1.5 times the maintenance should be instituted with isotonic fluids preferably 0.9% normal saline.
- In untransfused SCD patients, stroke recurrence rate is 67%, with 70% of recurrent strokes occurring in the first 3 years after the initial stroke [72]. As such, EBT should be followed up with hypertransfusion therapy to maintain sickle Hb level below 30% at a haemoglobin concentration of about 10 to 11 g/dL.
- Chronic blood transfusion (CBT) has been shown to be beneficial in primary and secondary prevention of CVD [70, 73–75]. However, clear definitions on when and how CBT should be stopped is yet to be made. Often times, transfusions continue till late adolescence or early adulthood.
- Hydroxyurea therapy reduces cerebral blood flow. Though less effective, hydroxyurea may be considered an alternative to chronic transfusion therapy, where transfusion is not feasible [67, 68].
- Thrombolysis with recombinant tissue plasminogen activator (rTPA) within the first 3 hours of ischaemic CVD in adult patients should be considered after careful patient evaluation [76]. TPA is not recommended in children. However, the current prospect of TPA use among Nigerian patients is remote due to challenges of its availability, cost, delayed diagnosis, and clinical experience with its use.
- Antiplatelet agent, aspirin 325 mg, is recommended if TPA is not used and should be avoided for the first 24 hours if TPA is used [77].
- Adult SCD patients should be evaluated and treated for modifiable risk factors such as dyslipidaemia.
- Acute stroke should be treated in a dedicated stroke unit with input of both neurologist and haematologist.

Haemorrhagic stroke tends to occur between 20 and 29 years of age and is associated with low steady state haemoglobin levels and high steady state leucocyte counts [71]. Hemorrhage often results from rupture of vessels within the circle of Willis. Clinical presentation is similar to ischaemic CVD. However, patients with haemorrhagic CVDs are more likely to present with coma. In haemorrhagic CVD, patient may present with severe headache, vomiting, and other features suggesting raised intracranial pressure. Haemorrhagic CVD is rare but more fatal. Cerebral oedema is worse in haemorrhagic stroke. Prompt confirmation of diagnosis through imaging studies is required. Treatment of cerebral oedema with hypertonic solution such as mannitol is desirable. Antiplatelet and anticoagulants are contraindicated in haemorrhagic stroke. Though its exact role in haemorrhagic CVD is not clear, EBT is recommended especially for patients billed to undergo magnetic resonance angiography [76]. Surgical interventions by vascular surgeons may include ligation of accessible aneurysms and surgical vascular bypass procedure for moyamoya syndrome. Nimodipine, a calcium channel blocker, improves outcome in adults with subarachnoid haemorrhage by counteracting delayed arterial vasospasm [76, 78].

Risk evaluation for an overt ischaemic stroke and the need for early preventive intervention are performed by assessment of cerebral blood flow velocity using transcranial Doppler (TCD) ultrasound. Cerebral blood flow in excess of 2 meters per second portends a high risk for CVD. Typically, TCD ultrasound assessment is commenced by age of 2 years. TCD between 1.7 and 2 should be reassessed in 3-4 months. If stable, assessment should be annual until 16 years of age [59]. Cerebral blood flow in excess of 2 meters/second is an indication for commencement of hypertransfusion therapy and this is shown to reduce stroke occurrence by about 90% [66]. MRI scan every 5 years may also be used in periodic evaluation of the brain for silent infarctions where resources are available [79].

Subclinical cerebral infarcts (SCI) in sickle cell disease patients occur in 27% and 37% of patients before their 6th and 14th birthdays, respectively [80]. Silent stroke is defined by an abnormal MRI in the absence of history and physical signs of an overt CVD. Risk factors for SCI include male gender, low steady state haemoglobin levels, higher baseline systolic blood pressure, and previous seizures [80]. Subclinical strokes are associated with neuropsychiatric dysfunction in apparently healthy SCD patients [79, 81] and are a risk factor for overt stroke [82]. In confirmed silent brain infarctions with neurocognitive delay and behavioural disturbances, chronic transfusion therapy is indicated.

#### 5.1.7. Acute Chest Syndrome (ACS)

ACS is a leading cause of mortality in sickle cell disease even among Nigerian patients, accounting for about 25% of all deaths [83–85]. Risk factors for acute chest syndrome includes older age, low fetal haemoglobin level, high haematocrit level, homozygous SS disease, chest VOC, smoking, general anaesthesia and surgery, asthma, and possibly opioid use [86]. ACS is defined by new pulmonary infiltrates (on chest radiography) in at least one complete lung segment, fever, and at least one respiratory symptom (pleuritic chest pain, cough, dyspnoea, and tachypnoea) [86]. ACS may follow a painful crisis especially in adults. ACS may also complicate the immediate postoperative state. As such, there is need to maintain proper protocols for preventing or treating ACS during BPC and after surgery.

The underlying pathophysiology of ACS includes vaso-occlusion of pulmonary vessels and microbial involvement. Implicated microbes include bacteria such as Pneumococcus,* Haemophilus influenza*, respiratory viruses, and atypical organisms such as* Mycoplasma*, Chlamydia, or* Legionella*. Respiratory viruses are more likely in children while bacterial causes are more frequent in adults [24]. Furthermore, hypoxia induced by ACS can trigger widespread sickling and vasoocclusion, with possibility of multiorgan failure and death. Fat laden pulmonary macrophages in the airways are observed in about half of the cases, suggesting possible contributions from bone marrow fat embolisation [83]. Bone pain crisis involving the chest cage can also trigger ACS as a result of pain induced hypoventilation, which encourages sickling in the pulmonary bed and microbial growth. Similarly, oversedation with opioids may predispose to ACS.

During BPC or in the immediate postoperative period, care should be taken to prevent ACS. In such patients, prophylactic incentive spirometry is helpful [87]. In the absence of a spirometer, 10 deep breaths every 2 hours of the day is an alternative. Treatment of an established ACS also includes incentive spirometry/chest physiotherapy, parenteral broad-spectrum antibiotics, effective pain relief, and supplemental oxygen therapy (2–4 liters per minute). Opioid is the mainstay of pain control in SCD. Its liberal use is encouraged in order to prevent pain undertreatment, prolonged treatment, and hypoventilation. However, care should be taken to avoid oversedation. Recommended antibiotic combinations include quinolones, 2nd or 3rd generation cephalosporin alongside macrolides (for atypical bacteria). Antibiotic choice should be further directed by local susceptibility profile if available.

Bronchodilator therapy is also required as most patients may have a bronchoreactive component. EBT is indicated in worsening lung consolidation or persistent hypoxia, any neurological deficit (confusion, motor deficit, epilepsy), intractable pain or opioid intolerance, haemodynamic instability, nosocomial infections, acute worsening of anaemia or cardiovascular insufficiency, and acute enlargement of spleen or liver [59]. Mechanical ventilation is required in rapidly progressive cases. Inhaled nitric oxide and steroid may be helpful in life-threatening cases. Evidence suggests that recurrent episodes of ACS can be prevented by hydroxyurea [88]. Also chronic transfusion therapy is beneficial in secondary prevention of ACS and is indicated in patients with two or more episodes annually, who are unresponsive to hydroxyurea [89].

#### 5.1.8. Priapism

Priapism is another acute complication of sickle cell disease. It is defined as persistent, purposeless, painful penile erection that is unassociated with sexual pleasure. Generally, reports of lifetime prevalence of priapism in sickle cell disease range from 2 to 35% [90]. Its prevalence is found to be as high as 44.9% among Nigerian male SCD patients [91]. Peak incidence occurs in 2nd and 3rd decade (median age: 18.5 years) [24, 91]. SCD priapism is a “low-flow” type. The penile ischaemia results from outflow obstruction (poor venous drainage) caused by sickled cells. Usually, it affects the corpora cavernosa alone while the spongiosum is spared. A typical genital examination reveals a hard penis with soft glans; tricorporeal involvement is rare. The priapism may also be defined as stuttering, minor, or major (prolonged) depending on the duration of the attack and its frequency. Stuttering priapism typically last about 30 minutes to 2 hours, tends to become recurrent (occurs several times a week), and may herald episodes of prolonged priapism. Minor attacks occur infrequently or isolated. Major or severe attacks last longer than 3 to 4 hours and should be treated as a urological emergency. Severe (major) and recurrent priapism (penile ischaemia) is associated with irreversible organ damage, fibrosis, and impotence.

The goal of treatment is to preserve erectile function and prevent recurrences. As such, there is need for early presentation in the hospital if home remedies are unsuccessful within 2 hours of onset. At the onset, patients should be counseled to drink extra fluid, use home-based simple or compound analgesia, and attempt to void. Other self-help strategies such as warm baths and gentle exercises like jogging may be helpful. Oral dose of pseudoephedrine or terbutaline may be given. If the priapism persists more than 2 hours, hospital care is required. This includes intravenous hydration and opioid analgesia. If the priapism persists more than 3 hours, aspiration and irrigation of the corpora with dilute phenylephrine, epinephrine, or etilefrine is indicated. Frequently, aspiration of blood from the cavernosal bodies is performed with a 23-gauge sterile needle, followed by irrigation with a 1 : 1,000,000 dilution of epinephrine in saline, after adequate counseling, conscious sedation, and local anaesthesia [92]. If detumescence is achieved lasting more than one hour, patient may be discharged home on oral analgesic, pseudoephedrine, and clinic follow-up. Penile aspiration and irrigation may be repeated up to 3 or 4 episodes if detumescence is not achieved early.

Simple early self-intracavernosal injection (SICI) of etilefrine and other adrenergic agonists such as metaraminol may achieve detumescence within one hour of onset, hence removing the need for hospital-based surgical aspiration and irrigation [93–95]. Sympathomimetics (adrenergic agonists) may be associated with untoward effects such as blood pressure changes and are yet to be licensed for SICI. EBT is indicated in recalcitrant cases. Surgical shunt procedures such as proximal shunt of quackel or distal shunt of winter may be tried, if conservative measures remain unsuccessful. However, surgical penile shunts may also be unsuccessful and may induce impotence [96]. Often, priapism will resolve with one or a combination of medical interventions.

In preventing priapism, male sickle cell patients ought to be adequately informed and counseled about priapism from adolescence. Patients with frequent episodes (≥2 per month, ≥4 per year) should receive priapism prophylaxis with oral pseudoephedrine 30 mg daily if they are less than 10 years of age and 60 mg per day if they are older than 10 years. Etilefrine and diethylstilbestrol (DES) may be used prophylactically although evidence for its usefulness is limited [97, 98]. DES use has been limited by its feminizing effects, though a short course of 5 mg daily may be used to abort a stuttering episode [99]. Similarly use of injectable leuprolide, a GnRH antagonist, which works through endogenous suppression of androgen production, is associated with longstanding hypogonadism and rebound priapism after discontinuation [97]. Use of hydroxyurea may also be beneficial [97]. There is recent evidence that use of phosphodiesterase (PDE) 5 inhibitor such as sildenafil is useful in preventing recurrent episodes of priapism [100–102]. Though not always successful, penile prosthesis may be remedial in those with established erectile difficulties persisting more than 12 months [103].

#### 5.1.9. Ocular Disease

Central retinal artery occlusion by sickled red cell sludge is an ocular emergency. It manifests as sudden change in vision. It is treated like stroke. Treatment requires EBT, hyperoxygenation, and reduction of intraocular pressure with carbonic anhydrase inhibitors [24]. Prognosis is however poor.

#### 5.1.10. Osteomyelitis

Osteomyelitis is one of the commonest skeletal complications of SCD [104, 105]. About 29% of Nigerian SCD patients experience this complication in their lifetime [106]. It often originates from bacteremia, as also observed in septic arthritis. Diagnosis may be quite difficult due to its similar presentation to acute bone infarction. Diagnosis requires a high index of suspicion. Serial blood cultures, as well as culture of local bone aspirates, may be required [59]. The commonest cause of osteomyelitis in SCD population is* salmonella *spp, followed by* staphylococcus *species [24, 107, 108]. Treatment requires involvement of the orthopedic surgeon and clinical microbiologist. Broad spectrum antibiotic based on the common local isolates and their susceptibility profile should be commenced after culture samples have been taken.

### 5.2. Chronic Morbidities in Sickle Cell Disease

#### 5.2.1. Delayed Growth and Development

Children with sickle cell disease have normal body weight at birth. However, by one year of life, there might be obvious weight lag when compared with normal infants. This weight deficit persists till adulthood and typically imparts a thin (asthenic) build [14]. Obesity may be seen in some cases [24]. Pubertal growth spurt may be delayed 1-2 years compared to their peers. Growth deficits in children with SCD may be due to multiple factors including severe anaemia, long-term effects of repeated vasoocclusion, endocrine failure, low dietary intake, and low socioeconomic status [109]. However, delay in skeletal maturation allows for bone growth such that final adult height is reached. Menarche may also be delayed for 1-2 years in females [24].

#### 5.2.2. Chronic Pain Syndromes

There are two forms of chronic pain in SCD: chronic pain due to obvious tissue damage such as AVN or leg ulcers, and intractable chronic pain with no obvious cause. Suboptimal treatment of recurrent severe acute painful crisis may progress to an intractable chronic pain syndrome. There is need for prompt and adequate treatment of acute pain episodes. Opioids coupled with nonopioids and adjuvant remain the mainstay of analgesia in SCD [45].

#### 5.2.3. Immunological and Infectious Complication

SCD patients have a subnormal immunity, which partly accounts for their increased susceptibility to infections [32]. Immunologic dysfunction in SCD is attributable to autosplenectomy with the resultant defective cellular and humoral immunity [110]. About 30% loss of splenic function occurs by first year of life and 90% by sixth year of life [111]. Normal splenic synthesis of immunoglobulins, properdin, and tuftsin is impaired, leading to increased susceptibility to infections. They are particularly susceptible to encapsulated organisms such as pneumococcus especially in children aged less than 5 years, hence the rationale behind pneumococcal vaccination and penicillin prophylaxis from four months of life till age five in western societies. Previous infections with* pneumococcus* confer lifelong prophylaxis. Studies reveal that, without preventive actions, invasive pneumococcal infection is 30 to 600 times more likely to occur in SCD children compared to normal persons [112].* Haemophilus influenza* is the next most common organism and affects children older than 5 years. In Africa,* Salmonella, Klebsiella*,* Escherichia coli*, and* staphylococcus* seem to be more common than* pneumococcus* [113, 114]. As such, routine prophylaxis against* pneumococcus* is not an established practice in Nigeria [115]. However, there is recent compelling evidence from other parts of Africa that* pneumococcus* contributes significantly to infections in SCD [116–118]. Since infection has been documented as the commonest cause of death among SCD patients in Nigeria, the role of pneumococcus in SCD related infections and mortalities needs to be clarified through further research. There is a need for vaccination and chemoprophylaxis against common infections [83]. Current national immunization schedule in Nigeria routinely includes vaccinations against polio, tuberculosis, Diphtheria, tetanus, pertussis, hepatitis B,* Haemophilus influenza* infections, measles, and Yellow fever. Before one year of life, the infant should have completed the vaccination schedule and is entitled to subsequent booster doses [119]. However, for persons affected with sickle cell disease, additional compulsory vaccinations should be administered to cover for* Streptococcus pneumonia*,* Influenza virus*, and* Neisseria meningococcus*,* human papillomavirus (HPV)*. Children less than two years of age should have four doses of the 7 valent pneumococcal vaccine between 2 and 15 months of life. The 23 valent pneumococcal vaccine should be administered at age of 2 years and older and should be repeated every 3 to 5 years till 10 years of age and every 5 years for those older than 10 years. Influenza vaccines should be administered during cold seasons beginning at 6 months of life. HIB vaccine should be commenced at 2 months of life. Meningococcal vaccination is recommended for patient at 5 years and older and is repeated every two years. HPV vaccine is administered to females under 26 years.

Also contributing to increased risk of infection in sickle cell disease is repeated tissue infarctions, which are potential foci for pathogens. Similarly, iron overload in patients that have had several transfusions favors growth of iron dependent bacteria such as* Yersinia enterocolitica*. Furthermore, micronutrient deficiency especially zinc deficiency is associated with lymphopenia and decreased immunity [120]. About 60–70% of SCD patients are zinc deficient. In Nigeria, a case-control study showed the serum zinc level to be significantly lower among SCD children compared with healthy controls [121]. Other studies have also shown significant deficiencies of other micronutrients such as magnesium and selenium [122, 123].

#### 5.2.4. Sickle Cell Chronic Lung Disease

Sickle cell chronic lung disease (SCCLD) is an age related morbidity. It affects at least a third of adult SCD patients [59]. Patterns of the lung involvement among Nigerian patients include restrictive lung disease, obstructive lung disease, chronic hypoxaemia, and pulmonary hypertension (PHT) [59, 124–126]. A Study in Nigeria reported a prevalence of 18.9% for SCCLD among adult SCD patients [127]. Chronic complications occur more frequently in those with history of acute chest syndrome. In about 20% of patients, echocardiography shows elevated pulmonary artery systolic blood pressure >35 mmHg. Incidence of PHT is higher in patients with high haemolytic rates and high LDH. PHT is associated with 10-fold increase in the relative risk of death and it confers poor prognosis [128]. Primary PHT is not the only cause of elevated TRV (tricuspid regurgitation jet velocity) in SCD patients. Patients with elevated TRV have increased risk of mortality in the next 3 years [59]. Treatment includes hydroxyurea therapy, chronic transfusion, vasodilator use, anticoagulation, and oxygen therapy.

#### 5.2.5. Hepatobiliary Complications

Chronic liver damage in sickle cell disease is caused by intrahepatic trapping of sickle cells, transfusion transmitted hepatotropic infections, and transfusion siderosis [59, 129]. Evidence suggests that posttransfusion hepatitis and other transfusion transmissible infections are still a significant problem in Nigeria [130, 131]. In Ibadan, Nigeria, Fashola and Otegbayo observed a posttransfusion viral hepatitis prevalence rate of 12.5% in 2002 [130]. In rare instances, vasoocclusion in the liver with cholestasis may precipitate acute liver failure. Pigment gallstones are found in about two-thirds of sickle cell patients, especially those with sickle cell anaemia [129]. Symptomatic gallstones require cholecystectomy. Cholecystitis is treated with antibiotics. Treatment of asymptomatic cholelithiasis may require watchful waiting. Gall stones associated with common bile duct stones require endoscopic retrograde cholangiopancreatography (ERCP). The risk of hepatic damage is reduced by ensuring viral safety of all transfused blood components and prompt institution of iron chelation therapy if iron overload is present. In hepatic failure, liver transplantation is a veritable option.

#### 5.2.6. Other Abdominal Complications

Incidence of peptic ulcer disease (PUD) is higher in SCD patients. PUD occurs in about 35% of SCD patients with epigastric pain [58]. In Ile-Ife, Nigeria, Akinola et al. observed PUD among 28% and 50% of Hb SS and Hb SC disease patients presenting with abdominal pains, respectively. Interestingly, duodenal ulcers are not associated with high acid outputs; rather, ulcers are secondary to decreased mucosal resistance, possibly due to bowel ischaemia and NSAID abuse.

#### 5.2.7. Renal Complications

The hypoxic, acidotic, and hypertonic state of the renal medulla favors vasoocclusion and destruction of the vasa recta. By the first year of life, SCD infants may develop hyposthenuria manifesting as nocturia or enuresis [24]. Local studies have shown that the prevalence of nocturnal enuresis is higher among children with homozygous sickle cell disease [132]. This further makes them susceptible to dehydration, especially in hot climate. In addition, distal type IV tubulopathy in SCD promotes acidosis, further predisposing to vasoocclusive events. Papillary necrosis (usually of the left kidney) presents with haematuria. Other possible causes of haematuria include infections, stones, and tumor. Recommended guidelines for treatment and prevention of sickle cell nephropathy (SCN) are presented as follows.

*Recommended Guidelines for Management of Sickle Cell Nephropathy*

- SCN is an age-related morbidity. Among Nigerian patients, its prevalence and severity increases with advancing age, longer survival, and homozygous SS disease [133, 134].
- Relevant clinical history and examination findings such as facial puffiness, loin pain, painless haematuria, leg and abdominal swelling, frothy urine, worsening anaemia, and hypertension, which may suggest renal disease, should be elicited at regular intervals during visits.
- At least once annually during maintenance visits, SCD patients should be assessed for their renal status. Recommended laboratory assays include urinalysis (on every visit), serum electrolytes, urea and creatinine (semiannually), creatinine clearance/estimated glomerular filtration rate (eGFR), and tests for microalbuminuria (albumin creatinine ratio, ACR; urinary protein to creatinine ratio, uPCR). A normal creatinine level does not exclude renal disease in SCD due to supranormal kidneys precipitated by hyperfiltration and increased secretion of creatinine and uric acid. Emphasis and therapeutic decisions should be placed on significant adverse changes in the renal markers rather than single absolute values.
- Consultations and comanagement with experienced nephrologist is recommended in the following setting: patients with uPCR >50 mg/mmol (442 mg/g), persistent microscopic haematuria, declining renal function (>10% fall in eGFR per annum), or eGFR <60 mL/min/1.73 m². Further evaluations including renal biopsy are necessitated in settings of sudden onset heavy proteinuria with or without nephrotic syndrome [135, 136].
- Treatment of haematuria includes bed rest, hydration, and blood transfusion if indicated in events of a significant blood loss. Most times, haematuria is caused by papillary necrosis. However, the possibility of a renal medullary cell carcinoma must be excluded in these patients.
- Progression of SCN to ESRD is often heralded by worsening proteinuria, anaemia, and hypertension. This may be delayed with adequate control of hypertension and proteinuria. Introduction of angiotensin converting enzyme inhibitors (ACEIs) or angiotensin receptor blockers (ARBs) reduces proteinuria [136]. For blood pressure control, diuretics are better avoided.
- Similarly, early commencement of hydroxyurea helps to delay progression to ESRD except hydroxyurea is contraindicated for other reasons.
- NSAIDS for pain control are better avoided in patients with established SCN, in order to prevent worse organ damage. NSAIDS cause significant decline in renal blood flow and glomerular filtration.
- Urinary tract infection in these patients should be treated aggressively. Patients with ESRD should be on regular EBT especially if renal transplant is being planned. End stage renal disease is managed with repeated dialysis, erythropoietin therapy, and/or renal transplant.

#### 5.2.8. Ocular Disease

Incidence of ocular disease is higher in Hb SC disease, Hb SB+thal compared to Hb SS [59, 137]. Repeated vasoocclusion in the vascular beds of the eye especially the retina causes progressive ophthalmopathy, which manifests as comma-shaped conjunctival vessels, iris atrophy, retinal pigmentary changes, and retinal hemorrhages. Neovascularization leads to sea-fanning, so called proliferative retinopathy. Eventually, vitreous haemorrhage and retinal detachment may occur. For prevention, annual eye examination is recommended for all SCD patients from the 2nd decade of life [59]. Treatment options for proliferative retinopathy include laser photocoagulation and vitrectomy.

#### 5.2.9. Sickle Cell Leg Ulcer

Leg ulcers are frequent in adults SCD patients especially males with SS phenotype and patients with low steady state haemoglobin levels [138, 139]. In a report from Benin City, Nigeria, Bazuaye et al. observed a prevalence rate of 9.6 and 22.4% for current ulcers and previous ulcers, respectively [139]. Ulcers commonly arise near the medial or lateral malleolus and may be single or multiple. The aetiology is often multifactorial and they include vasoocclusion of skin microvasculature, made worse by trauma, infection, warm climate, and iron overload [24]. Commonly isolated microbes include* pseudomonas aeruginosa*,* staphylococcus aureus*, and* streptococcus* species. Chronic SCD ulcers are painful and resistant to healing. Treatment of these ulcers requires multidisciplinary approach involving the haematologist, plastic surgeon, specialist nurses, and orthopaedic surgeon [24]. Generally, treatment includes pain relief (including local pain control before wound dressing), elevation of the leg, debridement (to remove necrotic tissue), elastic dressing/support bandage, and zinc sulphate therapy (600 mg/day). Some patients may benefit from chronic blood transfusion and skin grafting.

#### 5.2.10. Musculoskeletal Complications

Known musculoskeletal complications in SCD include medullary hyperplasia, dystrophic intramedullary calcification, H-vertebra, osteolysis, osteopenia, septic arthritis, dactylitis, ulcers, pathologic fracture, and osteomyelitis. H-vertebra or Cod-fish vertebra is due to infarction of the vertebral body, giving a fish-mouth appearance on radiography. In a study by Balogun et al., musculoskeletal complications occurred in 31.4% of adult Nigerian SCD patients [140]. Avascular osteonecrosis of the femoral and humeral head is particularly associated with reduced quality of life. AVN develops in about 50% of patients who survive to above 35 years of age and about 60% of patients who survive to 60 years of age [141]. Another recent study revealed that AVN occurred in about 13 per 1000 Nigerian SCD patients [142]. Exact mechanism for development of AVN is yet to be clearly described. Even patients with high fetal haemoglobin levels may not be totally protected from developing AVN. However, high steady state platelet count has been correlated with AVN in Nigerian SCD patients [142]. Other clinical and laboratory correlations of AVN include high haematocrit, coexistence of alpha thalassemia, and frequent VOC [143–145].

In older patients, humeral head necrosis is more common than femoral head necrosis, although femoral head necrosis is associated with more devastating pain due to weight bearing [146]. Treatment of musculoskeletal complications of SCD requires comanagement with an orthopedic surgeon with special interest in SCD. Persisting pain in a joint or at least a stage 3 arthropathy is indication for referral to an orthopedic specialist [146]. X-ray features of AVN may not be obvious until repair processes have changed the density of the bone. MRI is the investigation of choice in SCD patients with persisting hip or shoulder pain. Every patient with confirmed AVN should be staged with MRI. Initial conservative treatment should include counseling/patient education, analgesia, partial weight bearing on crutches, and physiotherapy. Option of joint replacement/arthroplasty is available for patients with severe joint destruction.

#### 5.2.11. Cardiovascular System Changes

Sickle cell disease is associated with cardiac abnormalities including dilated cardiomyopathy, ventricular hypertrophy, cardiac iron overload, dysrhythmias, pulmonary hypertension, myocardial infarction, and sudden death [147, 148]. Chronic anaemia in SCD potentiates ventricular hypertrophy and dilatation which may progress to left ventricular diastolic dysfunction and exercise intolerance [147]. Pulmonary arterial hypertension is defined by end systolic pressure in the right ventricle greater than 25 mmHg (normal is less than 15 mmHg). In a cohort of Nigerian SCD patients, 2 (3.6%) out of 56 met the criteria for pulmonary hypertension [125]. Tricuspid regurgitation jet velocity of >2.5 m/sec is associated with a high risk of pulmonary hypertension and is an independent risk factor for death [128].

#### 5.2.12. Transfusion Related Morbidities

Blood transfusion is a key therapeutic modality in SCD. In a cohort of Nigerian SCD children, the prevalence of blood transfusion is as high as 57% [149]. Benefits of transfusion in sickle cell disease include correction of the baseline anaemia, dilution of sickle haemoglobin levels, and suppression of endogenous sickle red cell production, as well as reduction in chronic haemolysis and circulating sickle cell levels [150–152]. Transfusion modalities in SCD include simple transfusions, exchange blood transfusion, or chronic blood transfusion (hypertransfusion). Simple transfusion refers to top up correction of anaemia. Indications for chronic blood transfusion include prevention of first stroke, prevention of repeat stroke, TCD USS >2 m/sec, delayed growth and development in children, frequent ACS, severe disease, severe SCD lacking HLA match, sickle chronic lung disease, pregnant women with bad obstetric history and frequent bone pains, and sickle cell leg ulcers [150, 151]. Indications for exchange blood transfusion include moderate to severe ACS, refractory painful VOC, stroke, central retinal artery thrombosis, and acute refractory priapism [36]. The choice of blood component for transfusion in SCD should be a sickle negative, recently donated (less than 7 days old), leucodepleted, and phenotypically matched for at least Rh and Kell antigens, racial and minority matched red cell concentrate. Cytomegalovirus (CMV) negative component should be used for transfusion in all CMV negative children, as they may be candidates for bone marrow transplantation. Target haemoglobin level should not exceed 10-11 g/dL in SCD as there are concerns for hyperviscosity and vasoocclusion [26, 153].

Transfusion of blood and blood components is not without risks. In particular, delayed haemolytic transfusion reaction and alloimmunisation are among the immunologic complications of blood transfusion associated with sickle cell disease. Due to their tendency for repeated transfusion from chronic prophylactic transfusion or otherwise, the risk of iron overload in body tissues with irreversible organ damages ensues, hence the need for close monitoring and prompt iron chelation when indicated. Reports on alloimmunisation rate among Nigerian SCD patients are lacking and may be related to the lack of routine alloantibody screening and extended red cell phenotyping in most blood banks in Nigeria.

Among nonimmunologic complications of transfusion therapy in SCD in Nigeria, transmission of viruses and iron overload is of note [89, 131]. Recent evidence suggests that transmission of viruses is still a major challenge to transfusion safety in Nigeria [131]. This calls for a better national transfusion service. SCD patients, particularly those on hypertransfusion therapy, are at particular risk for iron overload, with resultant damage to vital organs [89]. Iron status should be monitored in SCD patients, particularly those who have received a cumulative transfusion dose of more than 20 to 30 units. Chelation therapy should be instituted promptly if serum ferritin levels exceed 1000 ug/L [154].

#### 5.2.13. Psychosocial Issues/Psychiatric Complications

Psychosocial complications of SCD include poor self-image, negative thoughts and feelings about the condition, stigmatization, depression, cognitive impairments, fears, anxieties, hatred for parents and others, dropping out of school, and tendency for substance abuse [24, 155]. These complications are associated with the chronic nature, recurrent pain, reduced health related quality of life, and unpredictable course of the disease. Some degree of psychologic trauma is also rendered to parents and health caregivers. A recent report by Anie et al. revealed that about half of Nigerian SCD patients had depressive feelings [156]. Adequate psychological support should be provided for patients by physicians, other health care staff, parents, and support groups. Those requiring more definite intervention should be comanaged with clinical psychologists and psychiatrists.

A study in Jamaica revealed that 29% of SS patients had a psychiatric disorder, compared to 25% in the control population [157]. Association of psychiatric morbidity included leaving school early, difficulties in social adjustment, impaired cognition, and previous psychiatric difficulties [157]. Asthenic body builds and abnormal facies may be associated with poor self-image. Such patients should be identified early and treated. Other complications such as undereducation and underemployment may require the services of medical social worker and occupational therapy unit.

## 6. Special Care Situations

### 6.1. Sickle Cell Disease and Pregnancy

Typically, pregnancy in female SCD patients is attended by anaemia which may be worsened by pregnancy related plasma volume expansion and folate deficiency. VOC is more common in 3rd trimester [158]. Increased incidence of preeclampsia, maternal mortality, and perinatal complications such as abortions, stillbirths, low birth weight, and neonatal deaths are associated with SCD pregnancy [158–160]. As such, pregnant SCD patients require special care by specialists including experienced obstetrician, haematologist, midwives, and anaesthesiologist. Preferably, oral contraception should be recommended for sexually active SCD females and pregnancy should be planned. Folate supplementation should be ensured. A local study has shown that preconceptual care and early antenatal booking produce better outcome and less obstetric complications in SCD pregnancies [161]. Hypertransfusion is indicated in cases of bad obstetric history and severe sickle cell disease in pregnancy. SCD and pregnancy are procoagulant states. Coupled with obstetric surgeries, the risk of VTE is significantly increased and appropriate anticoagulation may also be necessary in their care-plan [45].

### 6.2. Perioperative Care in Sickle Cell Disease

Perioperative complications of surgery in SCD patients include hypoxia, dehydration, bone pain crisis, significant anaemia, and acute chest syndrome [162]. Anaesthesia may be associated with hypoxia and dehydration [163]. Good anaesthetic expertise and experience is indicated when undertaking surgical procedure in SCD patients [163]. Early surgical complications such as pain and haemorrhage should be well controlled. Optimal analgesia and tact surgical skills are indicated. Other strategies to improve perioperative outcomes in SCD include conservative preoperative blood transfusion therapy, epidural analgesia, and adequate postoperative pain control with opiate and nonopiate analgesia [164, 165]. Aggressive or exchange transfusion therapy has not been associated with better surgical outcomes [162, 164].

### 6.3. Sickle Cell Disease and Radiology

Infusion of radiologic contrast media may precipitate VOC. Hypertonic nature of contrast media triggers marked intracellular dehydration and marked increment in red cell MCHC, thus precipitating sickling. This complication may be averted by preprocedure red cell exchange to achieve target sickle haemoglobin level of 50%. Traditional iodinated contrast media (due to its high osmolality) are relatively contraindicated in SCD. Isotonic contrasts are safer to use in SCD [166].

## 7. Laboratory Diagnosis of Sickle Cell Disease

The science behind laboratory diagnosis of sickle cell disorder entails phenotypic testing for the presence the sickle haemoglobin and genetic analysis. Physicochemical properties of the sickle haemoglobin such as decreased solubility and sickling under deoxy conditions, its pattern of mobility in an electric field, and rate of elution from solution unto adsorbents are applied in its laboratory detection. Phenotypic tests may be used as screening tests or diagnostic tests. Screening tests chosen for the purpose of mass screening should be highly sensitive and cheap to run. Examples of screening tests include sickling test, solubility test, and alkaline haemoglobin electrophoresis. On the other hand, high specific, diagnostic tests include isoelectric focusing, citrate agar electrophoresis, and high performance liquid chromatography [167, 168]. Quantification of haemoglobin variants and globin chain studies are used in evaluation of compound heterozygous disease states such as sickle thalassemia syndrome [167, 169]. Hb A2 levels in excess of 3.5% are suggestive of haemoglobin S-beta thalassemia [168]. Other ancillary laboratory investigations useful in detection and monitoring of the disease include FBC, reticulocyte count, and peripheral blood film. Reticulocyte count usually range from 5 to 15% in sickle cell disease. On peripheral blood film examination, findings may include irreversible sickled red cells, polychromasia, occasional nucleated red cells, and schistocytes, as well as Howell-Jolly bodies [24, 111]. Target cells are seen in sickle haemoglobinopathies. In sickle cell thalassemia syndromes, target cells are seen alongside microcytes and moderate-severe hypochromia. Red cell indices may suggest macrocytosis due to increased reticulocytosis or compliance with hydroxyurea therapy. However, oval macrocytosis with hypersegmented neutrophils may suggest folic acid deficiency. Biochemical changes include high LDH, low haptoglobin, high total and indirect bilirubin, and high AST [35]. Genetic studies such as PCR are used for prenatal and preimplantation diagnosis [170].

## 8. Prognosis and Life Expectancy

Severe SCD is associated with poor outcomes, if no intervention is rendered. Known modulators of clinical severity include fetal haemoglobin levels, beta globin haplotype, amd coinheritance of alpha-thalassemia, as well as geographical and other unknown genetic factors [15, 16]. In a study by Emmanuelchide et al., a higher leucocyte count was associated with more SCD complications in a Nigerian SCD population [171]. Another recent Nigerian study in a cohort of 115 children with SCD showed the presence of dactylitis at first presentation and higher total WBC, neutrophil count, platelet count, and serum bilirubin levels to be significantly higher among those with severe disease, while a higher fetal haemoglobin level was associated with a milder disease [172]. Other notable poor prognostic factors include low haemoglobin F production, Hb less than 7 g/dL, Hb greater than 7 g/dL, high VOC rate, pulmonary HTN, and nocturnal hypoxaemia (more strokes) [59].

From a large cooperative study in USA in 1994, the median survival for SCA was reported as 42 and 48 years in men and women, respectively. For haemoglobin SC disease, it was reported as 60 years and 68 years for men and women, respectively [84]. In USA, 95% of children with SCD survive till adulthood [173]. In Jamaica, survival estimates for persons with SCA were reported as 53 years and 58.5 years for men and women, respectively [174].

Life expectancy in SCD is substantially reduced especially in those with severe disease. In a 10-year retrospective study reported in 2009 from Ilorin, Nigeria, by Chijioke and Kolo, the mean age of sickle cell anaemia patients was found to be 23 years compared to 40 years in the control population, suggesting reduced life expectancy [175]. Findings from that study also revealed that age correlated negatively with survival [175]. As recently reported by Ogun et al., the leading causes of mortality in Nigerian SCD patients include infections, acute chest syndrome, anaemia, acute sequestration crisis, and stroke. According to the study, the mean age at death was 21.3 years. Though some patients now attain fifth decade, most mortality occurs in their second and third decades of life [83].

## 9. Sickle Cell Disease Control and Current Challenges in Nigeria

Control of SCD begins with public education and definite strategies to prevent further transmission of the trait. Carrier detection and genetic counseling have been proven to be successful in curbing the spread of other haemoglobinopathies like thalassaemia [176]. Carrier detection should be offered at designated centres after proper genetic counselling through antenatal and newborn screening, couple/premarital screening, and other forms of population screening. Genetic counselling by trained personnel helps individuals at risk to take informed decisions about their reproductive life choices. The option of prenatal diagnosis and selective abortion in Nigeria is controversial and relatively unavailable. Local studies show that a significant proportion of Nigerians are averse to selective abortion, even if legally permitted [177–179]. Early detection and diagnosis of sickle cell disease is crucial to reducing mortality and mortality associated with sickle cell disease, as affected persons are offered early supportive and preventive treatments. Despite the huge burden of SCD, currently, there is lack of national or regional SCD newborn screening programme in Nigeria, as at the time of this publication. Specialized centers dedicated to care of SCD patients with requisite multidisciplinary teams and other facilities are grossly absent. Ideally, SCD infants diagnosed prenatally or through newborn screening should be routed to comprehensive SCD centers for optimal treatment [180]. Conversely, Nigerian SCD is still associated with delayed diagnosis [181].

Continuous training of healthcare professionals involved in care of SCD patients is also desirable. Further efforts should be directed at education of the patients and their parents or caregivers. The health caregivers should also constantly undergo professional refresher and update courses in order to optimize their knowledge and skills in care of SCD. Recent surveys still suggest a dearth of public health knowledge on sickle cell disease in Nigeria [182]. Despite Nigeria being the most populous black nation on earth with the highest burden of sickle cell disease, till now, there are no coordinated nationwide efforts aimed at controlling the disease. Current evidence suggests that the care available for patients with SCD in Nigeria is still suboptimal [183]. Secondary control measures such as chronic transfusion therapy and use of hydroxyurea are faced with peculiar challenges in developing nations such as Nigeria [89]. Such challenges include unavailability of blood and blood components, the need for patients and relatives to regularly source for blood and blood donors, cost of iron chelation, risk of transfusion transmissible infections, and overall cost of chronic blood transfusion [73, 89]. A recent study estimated the mean annual cost of hypertransfusion in Ibadan among paediatric SCD patient to be 3,276 US Dollars (SD = 1,168) [73]. Also, treatment of iron overload with metal chelators, which is a potentially inevitable complication of chronic transfusion, increases cost.

Furthermore, HSCT, which is the only potentially curative disease modifying intervention in sickle cell disease, is currently available in Nigeria and has been reported [171]. However, its practice is bewildered by ample challenges including poor government commitment, weak political will, poor infrastructure, unaffordability by the average eligible Nigerian SCD patient, lack of local bone marrow registries, absence of specialized molecular diagnostic laboratories, and epileptic electric power supply [184, 185].

## 10. Comprehensive Care in Sickle Cell Disease and Recommendations in Nigeria

Comprehensive care incorporates provision of holistic healthcare services including state-of-the-art diagnosis, standard therapies, preventive care, rehabilitative therapy, and other ancillary services, by a team of specialists in a given location, with maximum accessibility for all patients. Comprehensive sickle cell centers are grossly lacking in Nigeria. Holistic care has been shown to provide better outcomes in sickle cell disease evidenced by significant reduction in mortality, hospitalizations, and blood transfusion rates among Nigerian patients [186]. As well, WHO recommends that in areas where hemoglobin disorders are common, special dedicated centers with a high degree of autonomy are required in appropriate numbers and locations, with a high degree of autonomy [187]. Treatment of SCD requires a multispecialist team including professionals such as hematologist, pediatrician, orthopedic surgeons, plastic surgeons, ophthalmologists, nephrologist, specialist nurses, clinical psychologists, and social workers.

Provision of comprehensive health centers is crucial to improving SCD disease outcomes in Nigeria. At such facilities, treatment should be tailored to individual patient's needs. At diagnosis, proper education regarding the nature of the disease, possible complications, and its prevention and treatment should be offered to the patient and parents. Regular health maintenance visits should be scheduled and patients should be counseled on the need for adherence [188]. Compliance on the part of the patient depends on having adequate information on the disease and confidence in the health professionals. Similarly, timely and regular medical education should be provided to health care professionals involved in management of SCD in order to improve their expertise and skills. Also, establishment of support groups among patients is encouraged.

Comprehensive care centers must possess facilities for outpatient care, day-case admissions (day hospital services), and hospitalizations on a 24-hour basis [52]. Patients should have direct access (including phone contact) to such centers and their physicians. For acute complications and emergencies, a quick triage is carried out and prompt therapy is instituted. Standard protocols should be provided for management of specific complications, as well as general health maintenance. Scheduled review and strict adherence to protocols are advised.

Patients and parents should be counseled on avoidance of known precipitants of sickle cell crisis. Keeping a diary of pain episodes is helpful in identifying and avoiding triggers for pain crisis. Infections especially malaria have been reported as a major precipitant of sickle cell crisis among Nigerian patients. As such, vector control and chemoprophylaxis for malaria is recommended in all patients [189]. All forms of undue physical exertion or exhaustion should be discouraged. Mothers should be regularly reminded about routine national vaccination schedule as well as vaccination against organisms to which SCD children and adults are particularly susceptible, especially encapsulated organisms.

Adequate and regular hydration is important. At least 60–70 mL/kg of oral fluids or at least 1.5 L/m^2^every 24 hours is recommended [45, 187]. Hydration helps with haemodilution, which reduces the propensity for sickling and vasoocclusion. Regular hydration also prevents dehydration which they are prone to due to impaired concentrating ability of the kidneys. Exercise caution with fluid administration especially in those with renal disease or severe anaemia. Excessive fluids may precipitate pulmonary oedema and death.

Moreover, physicians should administer, monitor, and encourage patient's compliance with routine medications at follow-up visits. Routine medications include prophylactic antimalarial [190] and folic acid. Others may include antioxidants, aspirin, and prophylactic antibiotic (oral penicillin from 2-3 months of life until at least age 5 in areas where pneumococcal infection is prevalent). Malaria has been described as one of the major precipitants of VOC for patients in Malaria endemic regions including Nigeria, hence the rationale behind continuous life-long chemoprophylaxis [190–192]. However, according to a local study, no significant benefit or advantage was associated with routine chemoprophylaxis for malaria in SCD patients as both patients and controls had equal rates of asymptomatic parasitaemia and similar frequency of malarial attacks [193]. In Nigeria, the actual benefits of malaria chemoprophylaxis in SCD need to be clarified through further research.

Early institution of broad spectrum antibiotics is recommended in febrile SCD patients [187]. Antibiotic use should be guided by local bacteriological profile and should be commenced after necessary bacterial cultures are taken. A switch to appropriate antibiotic is based on sensitivity pattern of the offending isolate especially if the fever is persistent (unresponsive to the former antibiotic).

## 11. Disease Modifying Therapies

### 11.1. Hydroxyurea Therapy

Currently, hydroxyurea (HU) is the only approved disease modifying drug in SCD used for selected patients above 24 months of age [194]. HU is a cytotoxic agent that has been mainly used in treatment of CML and other myeloproliferative disorders. Its usefulness in SCD is related to its ability to induce increased levels of fetal haemoglobin production in sickle cells thus mitigating tendencies for red cell sickling. The exact mechanism is not fully understood, but, as a ribonucleotide reductase inhibitor, it prevents formation of deoxyribonucleotides, causing S-phase arrest of all replicating cells, thereby inducing stress erythropoiesis, which favors increased production of fetal haemoglobin [24]. HU is also known to increase steady state haemoglobin levels and reduce leucocyte and platelet counts. Also, as a rheological agent, HU improves cell hydration, limits interaction of the sickle cells with the vascular endothelium, and acts as a nitric oxide donor [195]. HU is of benefit to patients with moderate to severe sickle cell disease. Indications for HU therapy include recurrent VOC (3 or more severe episodes requiring admission in the last 12 months), recurrent ACS (2 or more episodes in a lifetime), severe symptomatic anaemia, and recurrent priapism, alternative to transfusion to prevent new or recurrent stroke especially where transfusion is not feasible [194]. Usually, HU therapy is commenced at 10–15 mg/kg once daily. Baseline investigations prior to commencement of hydroxyurea should include FBC, reticulocyte count, %Hb F, electrolyte urea and creatinine level, liver function test, uric acid, and LDH levels. Full blood counts are monitored weekly for the first 4 weeks, fortnightly for the next 8 weeks, and thereafter monthly if the counts remain stable [14, 194]. Its dose is increased by 2.5 to 5 mg/kg/day every 12 weeks (range of 4 weeks to 6 months) if absolute neutrophil count (ANC) >2000, Haemoglobin concentration >4.5 g/dL, and platelet count >80,000/*μ*L [14, 195]. As marrow suppression occurs, HU is withheld to allow for marrow recovery and then restarted at a dose of 2.5 mg/kg less than dose causing myelosuppression. This is known as the maximum tolerable dose [88, 195]. However, the ceiling dose for HU therapy is 35 mg/kg [151]. Minimum time interval for evaluation of therapeutic efficacy is 6 to 9 months [165]. Hb F levels should be monitored. HbF level in excess of 20% significantly ameliorates the disease. Complications of HU include myelotoxicity, mouth ulceration, macrocytosis and megaloblastoid changes, nausea, skin toxicity rashes, and hyperpigmentation [88, 194].

### 11.2. Haemopoietic Stem Cell Transplantation (HSCT)

Suggested eligibility criteria for HSCT in SCD include the following [196, 197]: (A) age <17 years; (B) at least one of the following complications: brain infarct/ischaemia (MRI), secondary cognitive impairment with cerebral vasculopathy, severe and recurrent ACS, ≥3 VOC per annum requiring hospitalization (>3 Hospitalisations for severe VOC in consecutive 3 to 4 years), moderate glomerular dysfunction, multiple epiphyseal aseptic necrosis, and grade I/II sickle chronic lung disease; (C) availability of HLA matched sibling donor. Exclusion criteria include donor with major haemoglobinopathy and one or more of the following: Karnofsky performance <70%, Portal fibrosis (moderate or severe), renal failure (GFR <30%), major intellectual impairment, stage III or IV chronic sickle lung disease, cardiomyopathy, or HIV infection. Older adults are considered less favorable candidates for HSCT due to the higher risk for severe organ toxicities and greater susceptibility to severe graft versus host disease [198]. HSCT should be performed in centers experienced in transplant for sickle cell disease.

### 11.3. Future Therapies

Aside from Hydroxyurea, other promising drugs that have been shown to modulate Hb F production but are still under investigation/trials include decitabine, 5-azacytidine, and short chain fatty acids such as butyrates [199, 200]. Other novel therapies are also being investigated. Their therapeutic efficacy is designed based on their targets against specific pathophysiological processes in SCD such as the abnormal membrane cation transport systems, increased/stimulated red cell-endothelial adhesiveness, endothelial activation and vasospasm, cellular dehydration, prooxidant state, and hypercoagulability in SCD. Gardos channel blockers such as clotrimazole and its analog, Senicapoc (ICA 17043), have been shown to reduce red cell dehydration and abate haemolytic rate and are well tolerated in SCD patients [201, 202]. Administration of magnesium salts is also observed to reduce red cell dehydration by inhibiting the KCL cotransporter. It is reported that infusion of magnesium sulfate reduced the length of hospital stay in patients with VOC [203]. However, this is not yet an established practice. Similarly, antiadhesive agents such as anti-P-selectin and heparin, as well as agents such as warfarin and aspirin for normalization of hypercoagulable state and Flocor for reduction of whole blood viscosity, and specific monoclonal antibodies for inhibition of red cell-endothelial adhesion are also being considered [199]. Inhalational nitric oxide and its precursor, L-arginine, are shown to be beneficial in acute vasoocclusive crisis and other ischaemic complications by increasing NO bioavailability [200, 203].

Theoretically, gene therapy offers a great hope of cure. However, effective vector for safe transfer and stable, erythroid specific expression of normal beta globin gene are still under investigation [204, 205].

## 12. Conclusion

Sickle cell disease is a major public health disease worldwide. There is still a high burden of the disease in Nigeria. There is still a significantly high rate of SCD complications and mortality among Nigerian patients. Current evidence suggests that available care is suboptimal. Largely speaking, prevention, control, and treatment of SCD in Nigeria are still in infancy. Yester efforts albeit present measures appear meager in the face of the enormous disease burden. There is need for a better coordinated effort towards control of SCD by the government at all levels and other concerned stakeholders. Appropriate interventional programmes backed by an effective national policy should be instituted. In addition, physicians involved in the care of SCD patients should be conversant with current knowledge and standard practices in the treatment of sickle cell disease in order to improve treatment outcomes.
